# NK cells protect TCR-transgenic mice from developing fetal leukemia

**DOI:** 10.1186/2051-1426-2-S3-P167

**Published:** 2014-11-06

**Authors:** Sigrid Dubois, Jürgen Müller, Lionel Feigenbaum, Thomas A Waldmann

**Affiliations:** 1 Lymphoid Malignancies Branch/CCR/NCI, Bethesda, MD, USA; 2 Transgenic Mouse Model Laboratory, LASP, NCI, Frederick, MD, USA

## 

To investigate the intrinsic effect of IL-15 expression on CD8 responses we generated IL-15-deficient OT1 TCR-transgenic mice. These mice died surprisingly at around six months of age exhibiting grossly enlarged lymph nodes, spleens and thymi. The affected organs harbored mainly CD8+ T cells that were low for MHC class I, expressed CD25 and CD24 as well as the co-stimulatory receptors CD28, ICOS and PD1. This phenotype resembled a sub-population of immature CD8 single-positive thymocytes. These **co**-stimulatory **r**eceptor-positive CD8 cells (CD8cor) expanded after transfers into mice that lacked NK cells due to IL-15 inhibition or antibody-mediated cell lysis, and NK cells caused in vivo lysis of CD8cor cells. In contrast, the presence of IL-15-dependent CD8+ T cells had no effect on CD8cor cell expansion. In vivo expansions of CD8cor cells also depended on the presence of CD11c-positive dendritic cells while IL-2 activity was dispensable despite high CD25 expression. These data suggest that NK cells prevent the thymic escape of a sub-population of CD8 single-positive thymocytes and their subsequent malignant transformation in TCR-transgenic mice.

